# Supplementary figures and images for: Wheat DOF transcription factors TaSAD and WPBF regulate glutenin gene expression in cooperation with SPA
Source: PLoS One. 2023 Jun 23;18(6):e0287645. doi: 10.1371/journal.pone.0287645 (PMC10289392; doi:10.1371/journal.pone.0287645)

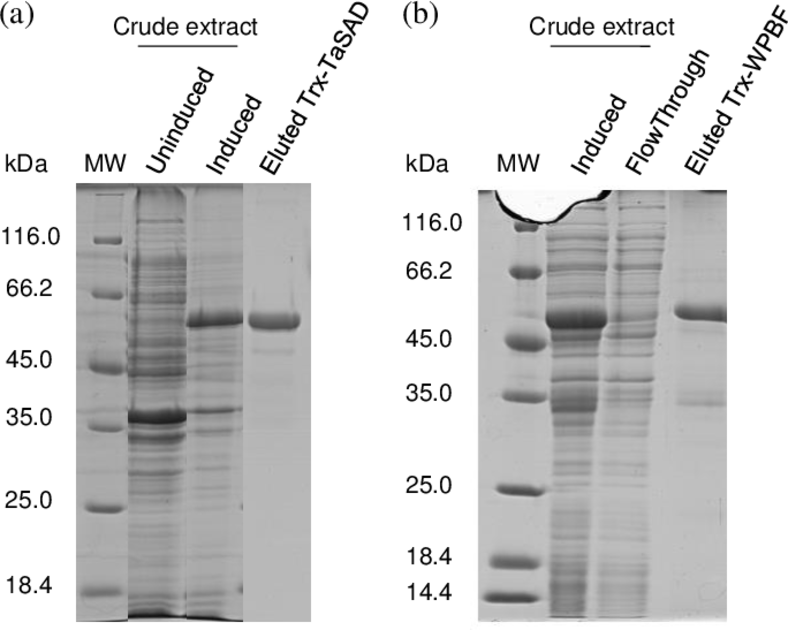

Supplement: S1 Fig — Expression and purification of recombinant proteins Trx-TaSAD (a) and Trx-WPBF (b). Crude extracts from uninduced or induced bacteria harboring the pET32Trx-WPBF and pET32Trx-TaSAD expression vectors and the proteins not retained (FlowThrough) or eluted during recombinant protein purification resolved by electrophoresis through a 10% SDS-polyacrylamide gel. MW: molecular weight markers, sizes are in kilodaltons (kDa). (TIF) [file pone.0287645.s001.tif]

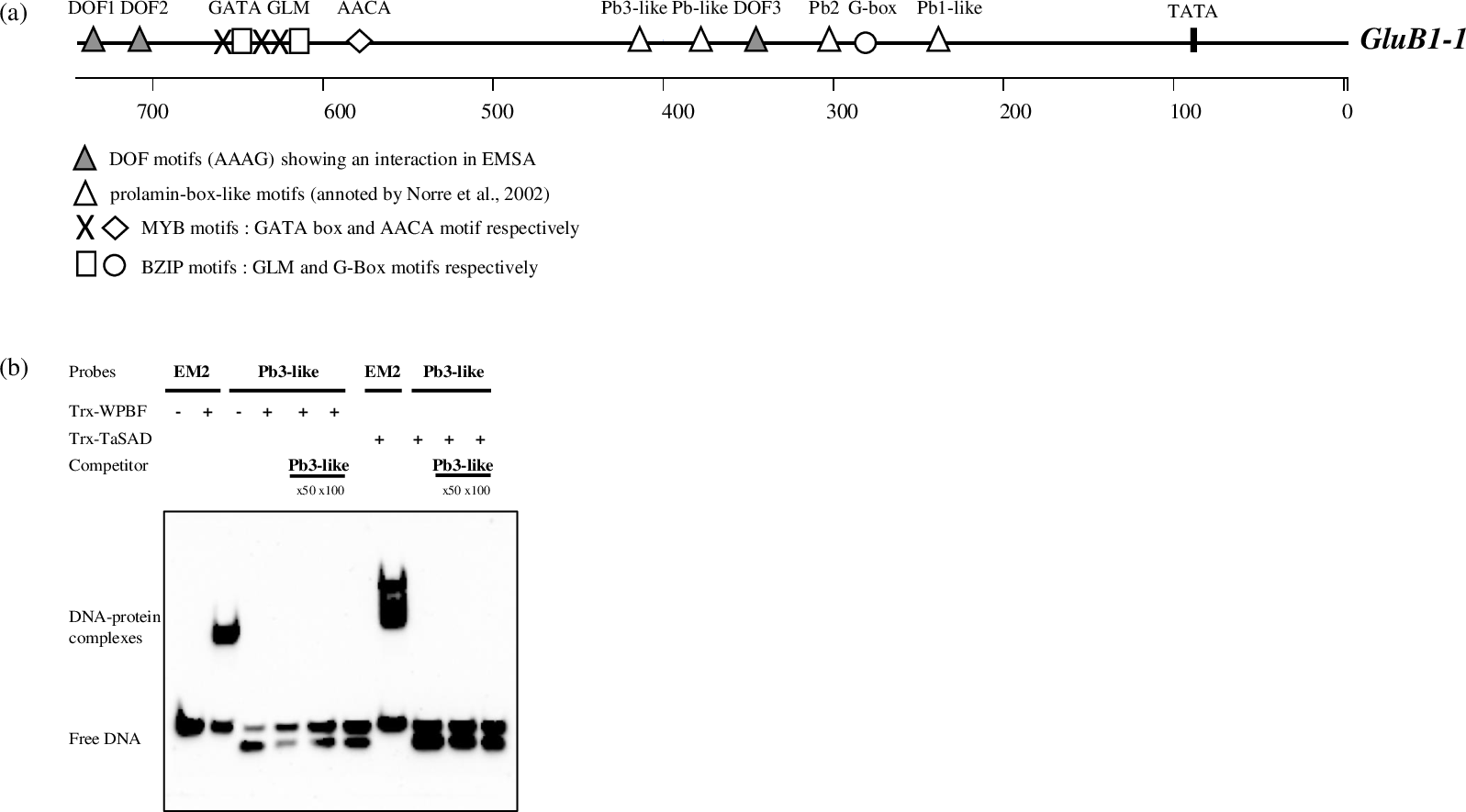

Supplement: S2 Fig — (a) Schematic representation of GluB1-1 promoter. The TATA box and nucleotide positions relative to the start codon are indicated. Putative cis-motifs, DOF1 (-742bp), DOF2 (-713bp) and DOF3 (-345bp), Pb3-like (-420bp), Pb2 (-314bp) and Pb1-like (-244bp), G-box (−280bp), GATA box (−661bp, −640bp, −636bp) and AACA motif (−580bp), GLM1 and GLM2 (−652bp and -631bp) are shown. (b) EMSA of recombinant Trx-WPBF (500 ng) and Trx-TaSAD (370 ng) proteins with the 22 bp biotin-labeled Pb3-like (-420bp) and EM2 (-337bp) probes derived from the GluB1-1 and GluD3 promoters respectively. (TIF) [file pone.0287645.s002.tif]
