# Supplementary material for: Wheat DOF transcription factors TaSAD and WPBF regulate glutenin gene expression in cooperation with SPA
Source: PLoS One. 2023 Jun 23;18(6):e0287645. doi: 10.1371/journal.pone.0287645 (PMC10289392; doi:10.1371/journal.pone.0287645)
Supplement: S1 Table — (DOCX) [file pone.0287645.s003.docx]

**S1 Table**. List of annotated DOF proteins used in phylogenetic tree analysis

| **Protein name** | **Species** | **Genebank number** | **References** |
| --- | --- | --- | --- |
| SAD | *Hordeum vulgare* | AJ312297 | Isabel-LaMoneda et al. 2003 |
| OsDof1 | *Orysa sativa* | AB028129 | Washio 2001 |
| OsDof2 |  | AB028130 |  |
| OsDof3 |  | AB028131 |  |
| OsDof4 |  | AB028132 |  |
| AtDof2-5 | *Arabidopsis thaliana* | AJ237810 | Gualberti et al. 2002 |
| AtDof3-7 |  | AJ224122 | Papi et al. 2000 |
| AtDof4-6 |  | AL035538 | Yanagisawa 2002 |
| BPBF | *Hordeum vulgare* | AJ000991 | Mena et al. 1998 |
| WPBF | *Triticum aestivum* | AJ012284 | Mena et al. 1998 |
| ZmPBF | *Zea mays* | U82230 | Vicente-Carbajosa et al. 1997 |
| ZmDof1 |  | X66076 | Yanagisawa and Izui 1993 |
| ZmDof2 |  | X79934 | Yanagisawa 1995 |
| ZmDof3 |  | X79935 |  |
