# Supplementary material for: Wheat DOF transcription factors TaSAD and WPBF regulate glutenin gene expression in cooperation with SPA
Source: PLoS One. 2023 Jun 23;18(6):e0287645. doi: 10.1371/journal.pone.0287645 (PMC10289392; doi:10.1371/journal.pone.0287645)
Supplement: S2 Table — (a) Housekeeping gene primers used for normalization of relative gene expression. (DOCX) [file pone.0287645.s004.docx]

**S2 Table.** Sequences of the primers used for qRT-PCR in cv. NB1.

| **Gene** | **Forward primer (5’–3’)** | **Reverse primer (5’–3’)** |
| --- | --- | --- |
| β-tubulin^(a)^ | CCATCAGTTGGTTGAGAATGC | CAAAGCTGGGAGTGGTCA |
| GAPDH^(a)^ | TTCAACATCATTCCAAGCAGC | CGTAACCCAAAATGCCCTTG |
| eF1α^(a)^ | CAGATTGGCAACGGCTACG | CGGACAGCAAAACGACCAAG |
| TaSAD | GCAACTCCACCAACACCAAG | GAGCGAGCCGCCCTC |
| SPA | AAGATGGCCGAGGAAACTATGAA | GGTTGGCAATGGATTATCACAGA |
| WPB | AGAAGAAGCCTCGGCCAAAG | CAGAACTTGGTGTTGCCAGACTT |
| HMW-GS | CGCTGAAGGTGAGGCC | CCTTGGGCGGCACCAC |
| LMW-GS | CAGCTAAACCCATGCAAGGTATTC | TGCGACCTAGCAAGACGTTGT |

^(a)^ housekeeping gene primers used for normalization of relative gene expression.
