# Supplementary material for: Wheat DOF transcription factors TaSAD and WPBF regulate glutenin gene expression in cooperation with SPA
Source: PLoS One. 2023 Jun 23;18(6):e0287645. doi: 10.1371/journal.pone.0287645 (PMC10289392; doi:10.1371/journal.pone.0287645)
Supplement: S3 Table — Probe sequences are shown with the cis-motifs in bold and mutated nucleotides in lowercase. (DOCX) [file pone.0287645.s005.docx]

**S3 Table.** Sequences of Prolamin-box, DOF, EM and mutated versions (*dof* and *em*) probes used in EMSA.

| **Probes** | **Forward (5’–3’)** | **Reverse (5’–3’)** |
| --- | --- | --- |
| Pb1-like | AATCTGTTT**TGCAAG**CACCAAT | ATTGGTG**CTTGCA**AAACAGATT |
| Pb2 | CACACTTC**TGCAAAC**AATACACCAG | CTGGTGTATT**GTTTGCA**GAAGTGTG |
| Pb3-like | GCATA**TTTGCGGAAG**CAATGGC | GCCATTG**CTTCCGCAAA**TATGC |
| DOF1 | GCC**AAAG**GTTTCAGTTAG | CTAACTGAAAC**CTTT**GGC |
| *dof1* | GCC**AgAt**GTTTCAGTTAG | CTAACTGAAAC**aTcT**GGC |
| DOF2 | TTGTCACGG**AAAG**GTGTTT | AAACAC**CTTT**CCGTGACAA |
| *dof2* | TTGTCACGG**AgAt**GTGTTT | AAACAC**aTcT**CCGTGACAA |
| DOF3 | AGATAA**AAAG**AACAGACCAAT | ATTGGTCTGTT**CTTT**TTATCT |
| *dof3* | AGATAA**cAgG**AACAGACCAAT | ATTGGTCTGTT**CcTg**TTATCT |
| EM1 | TGTGACAT**GTAAAGTTA**ATAAGGTGA | TCACCTTAT**TAACTTTAC**ATGTCACA |
| *em1* | TGTGACAT**cTAAAcTTA**ATAAGGTGA | TCACCTTAT**TAAgTTTAg**ATGTCACA |
| EM2 | CTCGGTT**GTAAAAGT**GATACTA | TAGTATC**ACTTTTAC**AACCGAG |
| *em2* | CTCGGTT**GTAgAAtTt**ATACTA | TAGTAT**aAaTTcTAC**AACCGAG |

Probes sequences are shown with the cis-motifs in bold and mutated nucleotides in lowercase.
